# Mycotic aneurysms as a rare cause of subarachnoid hemorrhage

**DOI:** 10.1038/s41598-025-20673-8

**Published:** 2025-09-23

**Authors:** Carolin Albrecht, Aminaa Sanchin, Ann-Kathrin Joerger, Claire Delbridge, Tobias Boeckh-Behrens, Nina Wantia, Silke Wunderlich, Lars Wessels, Peter Vajkoczy, Bernhard Meyer, Maria Wostrack

**Affiliations:** 1 Department of Neurosurgery, TUM School of Medicine and Health, Technical University Munich, Munich, Germany; 2 Department of Neurosurgery, Charité University Hospital, Berlin, Germany; 3 Department of Pathology, TUM School of Medicine and Health, Technical University Munich, Munich, Germany; 4 Department of Diagnostic and Interventional Neuroradiology, Technical University Munich, TUM School of Medicine and Health, Munich, Germany; 5 Department of Preclinical Medicine, Institute for Medical Microbiology, Immunology and Hygiene, TUM School of Medicine and Health, Technical University Munich, Munich, Germany; 6 Department of Neurology, TUM School of Medicine and Health, Technical University Munich, Munich, Germany; 7 TUM School of Medicine and Health, Technical University of Munich, Klinikum rechts der Isar, Ismaninger Straße 22, 81675 München, Germany

**Keywords:** Aneurysm, ruptured, Bacterial infections, Intracranial aneurysm, Mycotic aneurysm, Subarachnoid hemorrhage, Outcomes research, Predictive markers, Stroke, Cerebrovascular disorders

## Abstract

Aneurysmal subarachnoid hemorrhage (aSAH) caused by mycotic intracranial aneurysms (MIAs) is a rare cerebrovascular condition resulting from bacterial infiltration and arterial wall damage due to systemic or local infections. Due to limited data - often restricted to case reports or small series - no standardized diagnostic or therapeutic strategies exist. Therefore, this study aimed to characterize clinical, radiological, microbiological, and histopathological features of aSAH due to MIAs and to analyze treatment approaches in a larger cohort. We conducted a retrospective analysis of consecutive patients with aSAH from MIAs treated at two neurovascular centers between 2007 and 2024. Inclusion required both suggestive aneurysm morphology (e.g., distal, dysplastic) and evidence of systemic or local infection. Twenty-five patients (64% male, median age 44.7 years) were included. Mean Glasgow Coma Scale on admission was 8 ± 5, with most patients presenting with Hunt and Hess (HH) grades 5 (40%) or 4 (28%). MIAs were most often located at the middle cerebral artery (44%), with a median aneurysm size of 5.9 mm. More than one-third of patients had multiple aneurysms, and 16% developed new MIAs during hospitalization. Infections were associated with prior intracranial surgery (16%) or secondary foci, particularly infective endocarditis (44%). Hospital mortality was 36%, and the mean GOS at discharge was 2, highlighting the severity of the condition.

**Supplementary Information:**

The online version contains supplementary material available at 10.1038/s41598-025-20673-8.

## Introduction

Aneurysmal subarachnoid hemorrhage (aSAH) is a severe neurological condition associated with high mortality and long-term morbidity^1^. It poses a significant global public health challenge, with an estimated incidence rate of around 6.1 per 100,000 people per year^2^. Prehospital mortality ranges between 22% and 26%^3^, while inpatient mortality is reported to be around 20%^4^. aSAH occurs more frequently in women, and the incidence rate increases with age^1^. Among survivors, lasting cognitive deficits and permanent disabilities are common, accounting for the condition’s long-term impact.

Mycotic intracranial aneurysms (MIAs) are a distinct subtype of cerebral aneurysms that often lead to aSAH. Previous studies have reported a prevalence ranging from < 1% to – in rare cases – 5% of all intracranial aneurysms^5^. The development of these aneurysms is linked to systemic infections in which bacteria enter and damage the arterial wall. However, the term “mycotic” is not derived from the underlying fungal etiology but from the originally described mushroom-like appearance of the aneurysms^6^. Patients with ruptured MIAs are reported to be younger than those with typical aSAH, with most cases occurring in their third or fourth decade of life.

Based on previous findings, these aneurysms are mostly located in the middle cerebral artery (MCA)^5,7^. Recent studies indicate that MIAs predominantly grow in distal secondary and tertiary arterial branches due to septic emboli, frequently affecting the distal cerebral arteries. The underlying pathogenesis involves an inflammatory reaction that weakens the vessel wall, thus leading to aneurysm formation^8,9^. MIAs are typically described as irregular or fusiform in shape and relatively small^10^. However, when untreated, they may exhibit expansive growth, sometimes reaching several centimeters in size^11,12^. A notable finding in previous studies is the occurrence of multiple aneurysms in the same patient^11^. While multiple aneurysms are also seen in non-infectious cerebral aneurysms in approximately 15–35% of cases^13,14^, multiplicity appears to be a common finding in MIAs.

Histopathological examination of ruptured MIAs typically reveals transmural inflammatory infiltrates comprising neutrophils, lymphocytes, and macrophages accompanied by necrosis and thrombosis of the lumina^5^. A defining feature is destruction or fragmentation of the internal elastic lamina, with loss of medial and adventitial integrity^15^. Chronic or partially healed lesions contain granulation tissue and fibrosis. In some cases, pathogens are directly visualized within the wall of the aneurysm. Fungal hyphae (e.g. Aspergillus) or bacterial colonies can be identified using specific staining techniques^15^. These features are different to degenerative saccular aneurysms, which usually do not have active inflammation and have a normal structure of the vessel walls.

Endocarditis is a common source of infection, with studies suggesting that 2% of patients with endocarditis may develop MIAs^16^. Alternative sources of infection may include meningitis following previous neurosurgical interventions, orbital cellulitis, or mastoiditis^15^. In approximately 20 to 30% of cases, the origin of infection remains unidentified^5^. Although bacterial infections are most common, some patients may present with fungal or mycobacterial infections.

In addition, a large meta-analysis by Hamisch et al., which included 63 studies and 814 patients with ruptured MIAs, reported a mortality rate of 16.4%^17^. Of the 62 studies documenting treatment approaches, 56.1% used antibiotics alone, 20.9% combined antibiotics with surgery, and 23% used endovascular aneurysm closure.

Current evidence indicates that unruptured MIAs are frequently either resolved or reduced with appropriate antimicrobial therapy. However, data on MIAs are scarce and no therapeutic algorithm exists. Due to small sample sizes in the existing literature, there is little understanding of the devastating consequences and possible preventive strategies.

Therefore, the objective of this study was to provide a more detailed characterization of aSAH associated with the formation of MIAs, a rare and poorly understood clinical entity. Using a consecutive cohort of 25 patients treated at two large neurovascular centers, we aimed to systematically analyze clinical, radiological, microbiological, and histopathological characteristics; investigate therapeutic approaches, including surgical and endovascular strategies and assess functional outcomes and factors potentially influencing prognosis. To illustrate typical diagnostic and therapeutic challenges, a representative case is presented in detail.

The overall aim of this study is to improve our understanding of the heterogeneity and complexity of aSAH due to MIAs, with the goal of facilitating diagnostic measures and treatment decisions.

## Methods

### Literature review

We conducted a structured literature review using the MEDLINE database to identify previously published studies focusing on ruptured MIAs. The search strategy combined the terms: mycotic OR infectious AND cerebral aneurysm OR intracranial aneurysm AND subarachnoid hemorrhage. The search was restricted to studies published in the last 20 years. We included abstracts as well as full texts of case series (with more than one case), clinical studies and trials, as well as multicenter and observational studies. The language was restricted to English, and species limited to Humans; preprints were excluded.

Studies were eligible for inclusion if they documented more than one verified case of MIA and provided details on patient demographics, imaging findings, clinical presentation, and therapeutic management. Studies were excluded if they focused on extracranial mycotic aneurysms, lacked treatment strategies or did not offer accessible full-text versions. The titles and abstracts were screened for their relevance and selected articles were assessed in full. While systematic reviews were not the primary focus, some were considered to identify additional referenced cases or contextual insights.

### Study design and patient cohort

In accordance with STROBE guidelines^18^, we conducted a bicentric retrospective cohort study of patients treated for aSAH due to the rupture of MIAs from 2007 to 2024.

Patients were included to the analysis based on the following criteria:

1. Ruptured aneurysm confirmed by computed tomography (CT) angiography or digital subtraction angiography (DSA).
2. Evidence of local or systemic infection or sepsis in the patient that led to the formation of a suspected MIA.

We defined mycotic intracranial aneurysms (MIAs) as newly developed aneurysms occurring in direct association with a systemic or cerebral infection.

According to our diagnostic protocol, patients presenting with clinical deterioration and suspected aSAH initially underwent non-contrast CT and CT angiography. A digital subtraction angiography (DSA) was subsequently performed to confirm the diagnosis and further characterize the aneurysms. Treatment decisions were made by a multidisciplinary team involving neurosurgeons and neuroradiologists, allowing for a comprehensive assessment of each patient’s condition and the development of an individualized treatment plan. This approach was intended to balance the need for infection control with the management of the aneurysm to optimize outcomes for this cohort of high-risk patients. All cases were identified from the hospital databases by screening for the International Classification of Diseases, Tenth Revision (ICD-10) code for aneurysmal subarachnoid hemorrhage.

### Diagnostics

In general, both participating hospitals followed similar diagnostic protocols to identify the source of infection. These included urinalyses to rule out urinary tract infection or urosepsis, chest X-rays (or occasionally chest CT scans) to exclude pneumonia and blood cultures.

Transthoracic echocardiography (TTE) and transesophageal echocardiography (TEE) were performed in cases of suspected endocarditis. However, endocarditis diagnostics via TEE or TTE this was only performed in 60% of all patients.

Additionally, Ear-Nose-Throat (ENT) and maxillofacial examinations were performed to identify potential dental or ENT-related sources, such as otitis. HbA1c testing was conducted in patients with diabetes or those presenting with slow-healing wounds, such as leg ulcers. When the infection source remained unclear, an 18 F-FDG-PET scan was also considered.

### Outcome parameters

The analysis focused on identifying the primary source of infection and evaluating aneurysm characteristics, including size, location, and configuration, as well as associated clinical outcomes. By integrating this information with patient-specific factors such as comorbidities, we aimed to explore potential factors associated with poor outcome in aSAH due to MIAs. Furthermore, we analyzed therapy regimes regarding antimicrobial agents and the duration of treatments. In cases where intraoperative samples were submitted for histopathological evaluation, we retrospectively searched for characteristic inflammatory markers.

In terms of aneurysm location, distal aneurysms were defined as beyond the circle of Willis, on the anterior cerebral artery (ACA) distal to the anterior communicating artery complex (A2-A5 segments), on the middle cerebral artery distal to the limen insulae (M2-M4 segments), or on the posterior cerebral artery distal to the posterior communicating artery (P2-P4 segments)^19^.

### Ethical approval

This study was approved by the Ethics Committee of the Technical University of Munich, School of Medicine and Health (reference: 186/20S) and conducted in accordance with the principles of the Declaration of Helsinki. Given the retrospective design, the requirement for informed consent was waived by the committee. However, one recent representative case was selected in which intraoperative imaging of the aneurysm showed unique features. Medical records, imaging studies, laboratory results and operative reports were reviewed, and medical history was extracted from clinical documentation. As the patient remained incapable of giving consent, informed consent was obtained from his authorized family members in this specific case.

### Statistical analysis

Data analysis was conducted with GraphPad Prism (10.2.3, La Jolla, California, USA). For variables following a normal distribution, results are displayed as the mean ± standard deviation (SD). For variables that do not follow a normal distribution, descriptive statistics are provided as the median and range or, alternatively, as minimum and maximum values. Statistical significance between groups was evaluated using the student’s t-test or chi-square test for parametric data and the Mann-Whitney test for non-parametric data. Due to the limited number of patients and events, statistical analyses were restricted to univariate comparisons and correlation testing. The correlation was calculated using Spearman’s rank correlation. A significance threshold of *P* < 0.05 was applied to all tests.

## Results

### Literature review

Out of 112 records identified through the MEDLINE database search, 11 studies were included after screening of titles, abstracts, and full texts. These studies comprised a total of 125 patients with documented mycotic intracranial aneurysms. Although the treatment approaches varied widely, there was a clear trend towards interdisciplinary management. Endovascular techniques, most commonly parent vessel occlusion or coiling, were frequently employed and outcomes were mostly favorable with a low rate of periprocedural complications. Several studies have reported on strategies that combine surgery and endovascular treatment, particularly in cases involving multiple aneurysms or those with a more complex configuration. Across the studies, it was rarely sufficient to treat MIAs with antibiotic therapy alone. Spontaneous resolution was observed in only a minority of cases and failure or rebleeding during therapy was reported in multiple series. Overall, the findings support the active aneurysm exclusion to prevent rebleeding and improve outcomes.

All included studies are displayed in Table 1.

**Table 1:** Review of the literature (2005–2025) on aSAH due to MIAs.

Author(s)	Year	Title	Aneurysm multiplicity	Treatment approach	Main outcome/message
Nakahara et al.	2006	*Different modalities of treatment of intracranial mycotic aneurysms: report of 4 cases*	Four patients with five aneurysms	Medical only: one patient. Surgical clipping: one patient. Surgical trapping/resection (no bypass): one patient. Endovascular (coiling/trapping): one patient (treated for two aneurysms)	Prolonged courses of antibiotics are recommended for all patients with MIAs. Endovascular techniques (embolization or trapping) are effective and should be preferred for unruptured, dynamic mycotic aneurysms.
Kannoth et al.	2009	*Intracranial infectious aneurysm: presentation*,* management and outcome*	25 patients with 29 aneurysms	Medical treatment in 16 patients: 7 recovered (44%), 9 failed (3 died, 6 later required surgery). Early surgery in 5 patients: 1 death. Overall mortality: 32%	Clinical profile of MIAs varied significantly by infection source. Higher mortality was observed with: Meningitis-associated aneurysms, fungal etiology, vertebrobasilar location. Younger age was associated with better outcomes (*p* = 0.015).
Regelsberger et al.	2011	*Diagnostic and therapeutic considerations for “mycotic” cerebral aneurysms*	Two patients with one aneurysm each	Patient 1: antibiotics and surgical clipping with hematoma evacuation. Patient 2: endovascular coiling and 4 weeks of antibiotics	Early diagnosis via cerebral angiography and prompt antimicrobial therapy are critical. Surgical or endovascular aneurysm elimination is necessary.
Ota et al.	2012	*Ruptured infectious aneurysms of the distal MCA treated with trapping and STA-MCA bypass surgery*	Two patients with one aneurysm each	Surgical trapping and superficial temporal artery (STA) to MCA bypass surgery	STA-MCA-bypass is feasible. Neither patient experienced ischemic events postoperatively.
Esenkaya et al.	2016	*Endovascular treatment of intracranial infectious aneurysms*	15 patients with 17 aneurysms	13 underwent parent vessel occlusion, one coil occlusion, three occluded spontaneously	No perioperative neurological complications or aneurysm recurrences occurred.
Nonaka et al.	2016	*Endovascular Therapy for Infectious Intracranial Aneurysm: A Report of Four Cases*	Four patients with five aneurysms	Three parent artery occlusions, one direct aneurysm obliteration, one conservative	One case of aneurysm recanalization required re-embolization. All other had good outcomes.
Hamisch et al.	2016	*Interdisciplinary Treatment of Intracranial Infectious Aneurysms*	Six patients with six aneurysms	Antibiotics alone: 56.1%, Antibiotics and surgery: 20.9%, Antibiotics and endovascular therapy: 23.0%	Mortality rate: 16.8%. Antibiotic therapy is strongly recommended for all patients with MIAs. Surgical or endovascular interventions are recommended especially in ruptured cases.
Ohtake et al.	2017	*Initial treatment strategy for intracranial mycotic aneurysms*	Two patients with one aneurysm each	Patient 1: surgical trapping. Patient 2: conservative therapy (inoperable due to prior aortic root replacement)	Surgical intervention is preferred for both ruptured and unruptured MIAs. Antibiotics alone may reduce aneurysm size in unruptured MIAs.
Ando et al.	2019	*Treatment Strategies for Infectious Intracranial Aneurysms: Report of Three Cases and Review of the Literature*	Three patients with six aneurysms	Endovascular treatment for 5 aneurysms; 1 received combined endovascular and surgical treatment	Endovascular therapy preferred, but combined approaches warranted when either option alone is insufficient. Urgent treatment recommended for high rupture risk.
Rice et al.	2019	*Clinical course of infectious intracranial aneurysms (IIAs) during antibiotic treatment*	618 patients with infective endocarditis reviewed: 40 patients with altogether 43 MIAs	Initially treated with endovascular therapy: 18 (42%). Follow-Up without intervention: 25 (58%)	Only about 25% of MIAs resolved with antibiotics alone. MIAs diagnosed after longer prior antibiotic exposure were more likely to regress or resolve (*p* = 0.046).
Lee et al.	2020	*Bloodstream infection is associated with subarachnoid hemorrhage and infectious intracranial aneurysm in left ventricular assist device*	Three patients with four aneurysms	Patient 1: coil embolization and left ventricular assist device (LVAD) exchange, patient 2: antibiotics only; patient 3: transitioned to hospice	LVAD-associated subarachnoid hemorrhage (SAH) may be linked to MIAs.

### Patient characteristics

Between March 2007 and October 2024, 25 patients were treated for aSAH due to the formation of MIAs at both institutions. Of those, 64% were male and 36% were female. Median age was 44.7 years, with a range of 5–80 years. The overall incidence of MIAs in respect to all treated aSAH patients in the two centers was 1.03%. Patient demographics are displayed in Table 2.

**Table 2:** Patient demographics.

	Patient cohort (*n* = 25)
Age (median, range)	44.7 (5–80)
Sex	
– Male	16 (64%)
– Female	9 (36%)
HH grade (median, range)	4 (2–5)
Fisher grade (median, range)	4 (3–4)
GCS at admission (median, range)	9 (3–15)
Treatment	
– Clipping	14 (56%)
– Endovascular	7 (28%)
– Conservative	4 (16%)
Aneurysm location	
– MCA	48%
– ACA	16%
– ICA	8%
– PCA	8%
– Other	20%
Aneurysm size (mean, SD)	5.9 mm (3.2. mm)
Multiple aneurysms	9 (36%)
New aneurysm formation	4 (16%)
Number of aneurysms (median, range)	2 (1–25)
External ventricular drainage (EVD)	18 (70%)
Hydrocephalus with VP Shunt implantation	10 (40%)
Cerebral Vasospasm (CVS)	2 (8%)
– Endovascular spasmolysis	1 (4%)
Prior intracranial surgery	4 (16%)
HIV	3 (12%)
IV drug abuse	4 (16%)

None of the patients had a history of aSAH in the past. Among all, four (16%) patients had prior intracranial surgery. Intravenous drug abuse was reported in 20% (*n* = 5) of the patients and HIV was present in three patients (12%), two of whom were not receiving antiretroviral therapy.

In total, four patients originated from non-European countries (India, Russia, Saudi Arabia, and the United Arab Emirates), whereas the remaining 21 patients were from European nations.

**Fig. 1:**
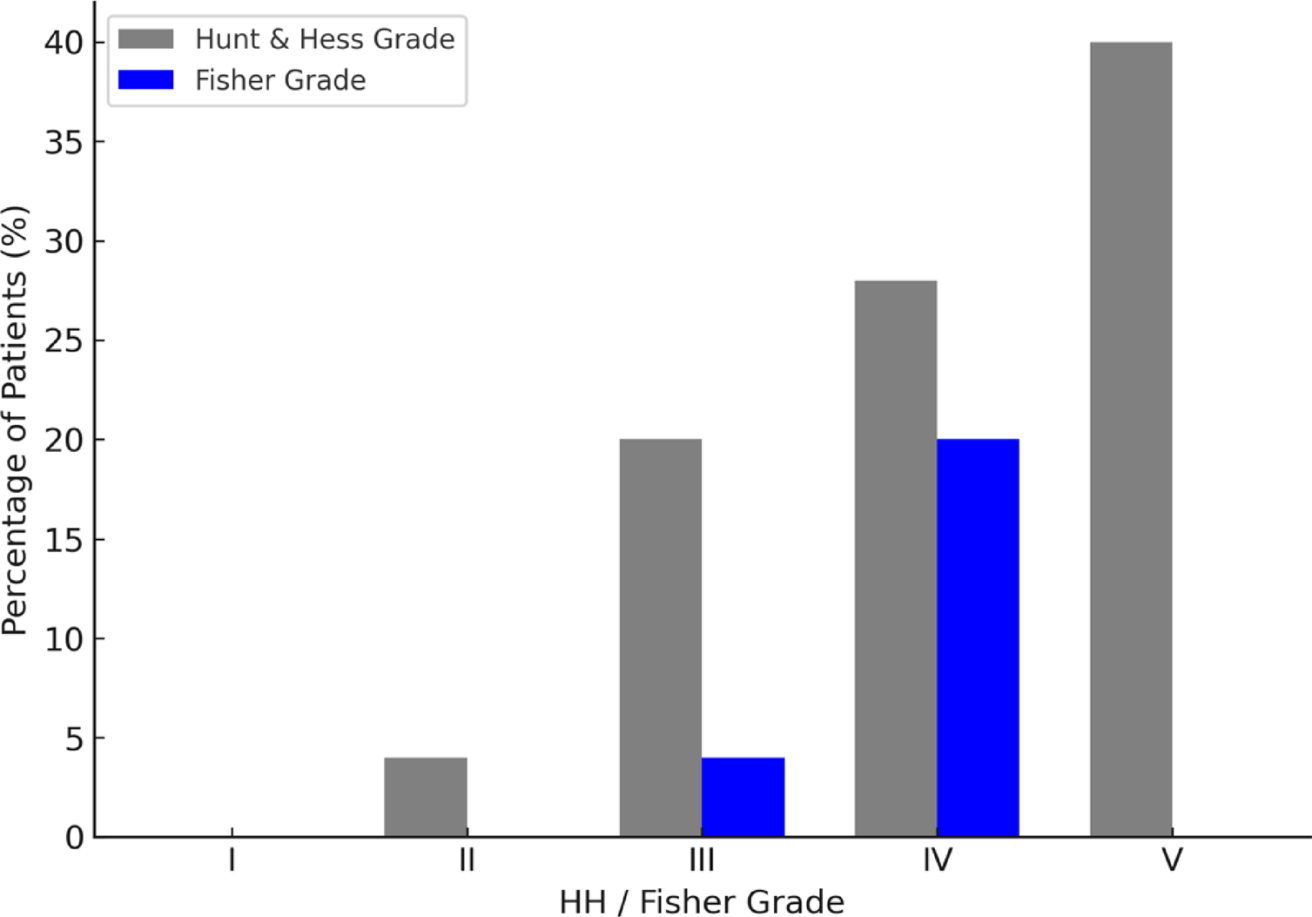
Distribution of HH grades and Fisher grades at admission. Most patients presented with high-grade hemorrhages (HH IV–V and Fisher IV), indicating severe initial clinical and radiographic status. These findings highlight that most mycotic aneurysm ruptures were associated with severe subarachnoid hemorrhage and poor clinical condition at presentation.

Mean GCS at admission was 8 (± 5). Seven patients presented with Hunt & Hess (HH) grade 4 (28%) and ten patients with HH grade 5 (40%), whereas five were classified as HH grade 3 (20%) and one as HH grade 2 (8%, Fig. 1). In two additional patients, HH grading was not applicable: one was already unconscious at the time of the aSAH, which was made due to massive blood reflux through the external ventricular drain (EVD). The other patient was a 5-year-old child with nonspecific symptoms, in whom the aSAH was detected incidentally during imaging performed to exclude cerebral embolism in the context of suspected endocarditis.

In two patients (8%), the diagnosis of the presence of a MIA was made before the onset of aSAH during the same hospitalization. Eighteen patients (72%) presented with intraparenchymal hemorrhage. Distribution of Fisher grades on initial CT scans is displayed in Fig. 1.

### Aneurysm occlusion and further treatment

Of the 25 patients, 14 (56%) underwent microsurgical clipping, including one case in which prior endovascular treatment had failed, while seven patients (28%) were successfully treated endovascularly. Four patients (16%) received antibiotic therapy only. In all cases, the decision against procedural treatment was based on severe clinical instability, poor prognosis, or an exceptionally high procedural risk. One patient presented after prior treatment for basal meningitis and ventriculitis and was already in a comatose state with brainstem ischemia, precluding further intervention. Another patient suffered a massive aSAH with cerebral edema and signs of herniation following treatment for a known intracranial abscess; due to the critical radiological findings, only supportive care was provided. A pediatric patient with endocarditis and severe cardiac comorbidities (mitral valve prolapse and insufficiency) was considered too unstable for neurosurgical or endovascular therapy. The fourth patient was diagnosed with a total of 25 aneurysms and deteriorated rapidly, dying before any intervention could be initiated.

One patient was initially scheduled for endovascular treatment, but when this approach was unsuccessful, the patient was immediately transferred to surgery. Another patient underwent elective intracranial bypass surgery and developed aSAH one day postoperatively due to an ineffective bypass.

Surgical approaches were preferred in cases of superficial aneurysm location like MCA aneurysms, intraparenchymal hemorrhage with mass effect requiring treatment or failure of endovascular options (*n* = 1). Endovascular therapy was chosen for deeper or more distal aneurysms, or for patients who were considered poor surgical candidates.

Further treatment included hemicraniectomy in 32% of patients to treat increased intracranial pressure (ICP) and brain swelling identified on CT scan following ICP elevation. Of two patients diagnosed with cerebral vasospasm by daily transcranial Doppler (TCD) monitoring, only one required endovascular spasmolysis due to neurological deterioration. In addition, 18 patients (70%) received an external ventricular drainage (EVD) of whom 10 patients (40%) required permanent CSF drainage in the form of a ventriculoperitoneal shunt (VPS).

### Aneurysm configuration and clinical data

In most cases, the aneurysm was located in the middle cerebral artery (MCA, 48%), followed by the anterior cerebral artery (ACA, 16%). The internal carotid artery (ICA) and the posterior cerebral artery (PCA) were each involved in 8% of cases. The superior cerebellar artery (SUCA), posterior communicating artery (PCOM), posterior inferior cerebellar artery (PICA) and basilar artery (BA) were each involved in 4% of cases. Digital subtraction angiography (DSA) was not performed in one case of emergency clipping and two cases of early death.

**Fig. 2:**
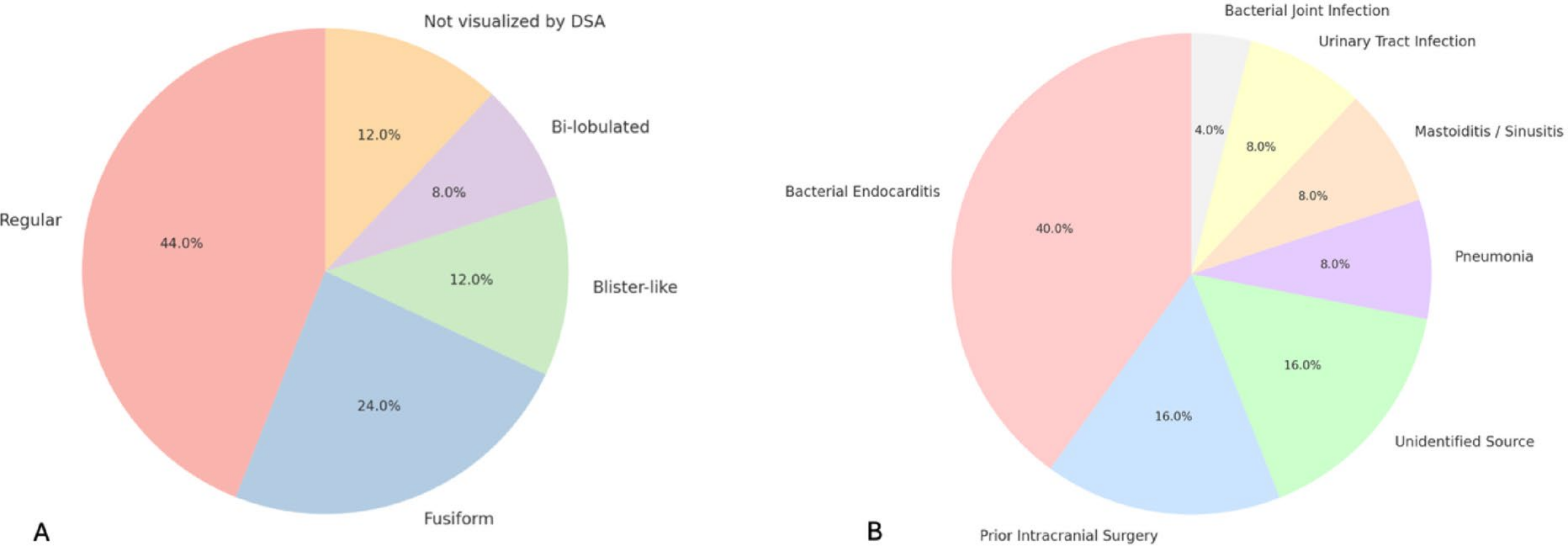
(**A**) Pie chart showing aneurysm morphology in all patients. *Note*: Fusiform configuration accounts for all irregular aneurysms that are not categorized as bi-lobulated or blister-like. (**B**) Pie chart showing infection sources. Mycotic aneurysms present with a wide range of morphologies. Bacterial endocarditis remains the most common underlying infection.

Mean size of the MIAs was 5.9 mm (SD 3.2 mm). Eleven aneurysms (44%) had a regular saccular configuration, while six were described as fusiform or irregular (24%), three as blister-like (12%), and two as bi-lobulated (8%, Fig. 2 and A).

Three aneurysms (12%) were located at bifurcations, while 17 aneurysms (68%) were found in distal locations such as the M3-branch (24%).

Nine patients (36%) had multiple aneurysms on the first DSA, with a median of two aneurysms (range 1–25). Additionally, four patients (16%) developed new aneurysms during the hospital stay. Rebleeding occurred in three patients (12%) with one patient suffering from overall five bleeding events.

### Primary infection site and infection markers

In line with previous evidence, endocarditis was the most common underlying source of infection (Fig. 2 and B). Among the 25 patients, 10 (40%) were diagnosed with bacterial endocarditis. Other identified sources included pneumonia in two patients (8%), mastoiditis/sinusitis in two cases (8%), urinary tract infections (UTI) in another two patients (8%), and a bacterial infection of the joints in one case (4%).

In one of the two patients with pneumonia, concurrent bacteremia was confirmed. In the second case, although blood cultures remained negative, the diagnosis was supported by clear radiological findings of pulmonary consolidation, markedly elevated inflammatory markers, and the absence of any alternative infectious focus.

Among the two patients with UTI, one had positive blood cultures for Escherichia coli, and histopathological examination of intraoperatively obtained aneurysm tissue revealed inflammatory wall changes consistent with a mycotic etiology. In the other, bacteremia with Streptococcus agalactiae, Klebsiella pneumoniae, and Proteus mirabilis was detected.

However, especially in the pneumonia case without confirmed bacteremia, the association with aneurysm formation remains presumptive.

In 16% of cases presented with proven bacteremia, the source of infection remained unidentified despite thorough diagnostics, including whole body 18 F-Fluorodeoxyglucose Positron Emission Tomography (18 F-FDG PET) CT scan.

Four patients (16%) had prior neurosurgical intervention. Median duration between the surgery and the detection of the MIA was 35 days (IQR 5 days).

In three out of four cases (75%), aneurysm growth occurred at the surgical site after prior intracranial surgery for another entity. In the fourth case, aneurysm detection was not possible due to the patient’s early death. Infection markers were not specific. However, eleven patients (44%) showed signs of sepsis at the time of the bleeding. These were hypotension and catecholamine dependency in 77.8%, organ dysfunction in 44.4% and respiratory insufficiency in 22.2% of cases. Additional elevated PCT levels were present in 33.3%, leukocytosis in 11.1% and thrombocytopenia in 11.1%.

### Intraoperative histopathological and Microbiological results

Of the patients who underwent clipping, 56% had samples sent for histopathological analysis and 40% for microbiological analysis. Of these samples, six (24%) contained aneurysm tissue.

Histological samples showed infectious changes in all cases, while two cases clearly showed marked inflammatory destruction of the vessel walls. Eight other probes contained organized hematoma in four cases, brain parenchyma in two cases, aspergilloma in one case and thrombotic material in another case.

Histopathologic analysis revealed distinct and consistent features of infectious vascular injury across samples. A hallmark finding was the infiltration of the aneurysm wall by acute and chronic inflammatory cells, predominantly neutrophils, lymphocytes, mononuclear cells and plasma cells. This inflammatory response was often accompanied by disintegration of the vascular wall architecture. In contrast to non-infectious aneurysms, the extracellular matrix in our specimens appeared markedly altered, with regions of pronounced irregularity, damage, and necrosis indicating advanced tissue breakdown. In several samples, Elastica van Gieson (EvG) staining revealed fragmentation and focal loss of elastic fibers within the tunica media and adventitia. The media itself appeared thinned and discontinuous. PAS staining showed dense inflammatory infiltration, especially in the thickened aneurysm wall, with scattered nuclear debris and disrupted matrix architecture.

Overall, in 16 of the 25 patients (64%), at least one pathogen could be identified by microbiological culture in either intraoperative tissue or blood samples, or both. In three cases, a fungal pathogen was detected (Table 3). Seven patients with positive pathogen detection (28% of all patients) had only a single pathogen, while the remaining nine patients exhibited mixed microbial flora. *Abiotrophia defectiva*, a rare but clinically relevant cause of infective endocarditis was identified in one patient with clinically prevalent endocarditis.

**Table 3:** Pathogens detected in all patients.

	Pathogen	Count
**Gram-Positive Cocci**	*Staphylococcus aureus*	3
	*Staphylococcus hominis*	1
	*Staphylococcus epidermidis*	4
	*Staphylococcus saccharolyticus*	1
	*Streptococcus pneumoniae*	1
	*Streptococcus mitis*	1
	**Group A**: *Streptococcus pyogenes*	1
	**Group B**: *Streptococcus agalactiae*	1
	**Anginosus Group**: *Streptococcus anginosus*	1
**Gram-Positive Rods**	*Cutibacterium acnes (C. acnes)* *Gemella haemolysans*	4 1
**Gram-Positive**,** Nutritionally Variant Streptococci (NVS)**	*Abiotrophia defectiva*	1
**Gram-Negative Enterobacterales**	*Escherichia coli (E. coli)*	1
	*Proteus mirabilis*	1
	*Klebsiella pneumoniae*	2
**Non-Lactose Fermenter**	*Pseudomonas aeruginosa*	1
**Fungi**	*Candida albicans*	2
	*Candida parapsilosis*	1

The table includes both intraoperative samples and blood culture specimens.

In one of the three HIV-positive patients, *Streptococcus pyogenes* and *Staphylococcus aureus* were detected, while *Staphylococcus aureus* was also identified in a second patient. No pathogenic microorganisms were isolated in the third patient. Additionally, two of the three patients had a history of intravenous drug use.

As described in the literature, most pathogens detected were Gram-positive bacteria, such as *streptococci* and *staphylococci*, which are predominantly found in the oral mucosa and skin. Notably, four Gram-negative pathogens were also identified which have been reported in the literature to cause mycotic aneurysms although they are rarely associated with cerebral mycotic aneurysms. *Salmonella spp*. and *Campylobacter spp*. were not found in our study population.

In total, *Cutibacterium acnes* (*C. acnes*) was detected in four patients (Supplementary Table 1). While often considered a contaminant in blood cultures it is known to play a significant role in prosthetic valve endocarditis and prosthesis-associated infections. The role of *Cutibacterium species* in this context remains unclear. However, in our case report, *C. acnes* were found in two blood cultures and in the aneurysm biopsy, suggesting a possible etiological role of *C. acnes* in this condition.

Among the three patients in whom fungi were detected, one was an active IV drug user, while the other two showed no signs of immunosuppression. Out of the total 25 patients, five were classified as immunocompromised: three had HIV (two of whom were also active IV drug users), one had B-cell non-Hodgkin lymphoma, and one was an IV drug user without HIV (Supplementary Table 2).

### Antibiotic and antimycotic regime

Information on antimicrobial therapy was available for 21 of the 25 patients. The duration of intravenous antibiotic treatment ranged from 1 to 160 days (median: 35 days).

The choice of the antimicrobial agent was adapted to the pathogen identified. If a specific pathogen could not be isolated, antibiotic therapy was given according to the internal guidelines of our microbiology team. In cases of postoperative intracranial infection, vancomycin and meropenem were usually applied. Altogether, vancomycin was the most common antibiotic agent and was used in 15 patients (60%). In 13 cases (52%) it was administered in combination with meropenem. In one patient, in whom the source of infection remained unidentified, and no pathogen was detected, a combined therapy including metronidazole, ceftriaxone and flucloxacillin was applied due to an assumed native intracranial infection.

Among 16 surviving patients, data on further antibiotic treatment was available for 12 patients (75%). Of these, four received oral antibiotics and two continued to receive intravenous antibiotics in parallel. Altogether, only two patients were transitioned to oral antibiotics alone, receiving them for 6 and 8 weeks, respectively.

**Fig. 3:**
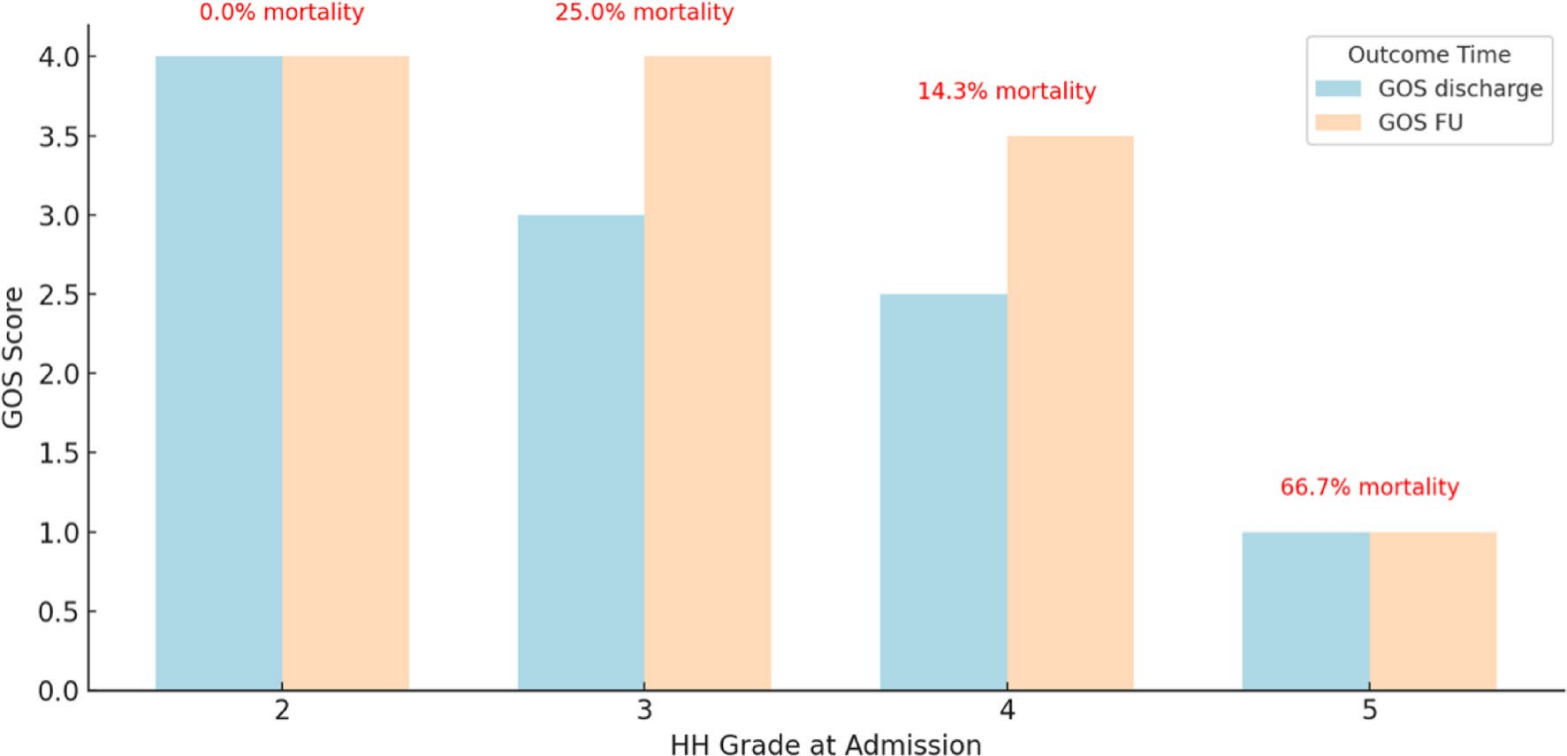
GOS at discharge and Follow-Up by HH grade at admission, showing that higher HH grades were associated with worse outcomes and increased mortality. This underscores the prognostic value of initial clinical severity in ruptured MIAs.

Among all patients, nine (36%) received antimycotic therapy. This was the case, when fungal infections were suspected, or prophylactic antifungal measures were considered. The median duration was 14 days (IQR 10 days). A fungal pathogen could be proven in three instances (12%, Fig. 3). In that cases, antifungal therapy was prolonged for a median of 40 days.

### Outcome and predictive factors

The outcome was overall poor. Nine patients (36%) died during the hospitalization. Of the 16 survivors, the mean GOS at discharge was 2 (± 1) with follow-up data available for 13 patients (52%), whereas the mean time to follow-up was 8.2 months (± 8 months). During the follow-up, the surviving patients exhibited limited recovery, with a mean GOS of 3 (± 1).

Initial HH grade was a significant predictor for the outcome whereas higher HH grades at admission were associated with lower GOS at discharge (*p* = 0.02, Spearman’s correlation, Fig. 3). However, there is no statistically significant association between HH grades at admission and survival (*p* = 0.135, Fisher’s exact, Supplementary Fig. 1).

Fisher CT grades, as well as GCS scores at admission, did not correlate with outcomes measured by GOS in our cohort (*p* = 0.845 and *p* = 0.23, respectively, Spearman’s correlation).

Furthermore, the presence of intracerebral hemorrhage did not significantly correlate with GOS (*p* = 0.954, Spearman’s correlation).

Regarding treatment outcomes, the use of antimycotic agents or targeted antimicrobial therapy was not associated with higher GOS at discharge (*p* = 0.172, Table 4; Supplementary Fig. 2). In a subset of 18 patients with complete data of antibiotic treatment, a significant positive correlation was observed between longer antibiotic treatment and higher GOS at discharge (Spearman’s *r* = 0.50, *p* = 0.033). However, in the subgroup of surviving patients (*n* = 16), this trend did not reach statistical significance (*p* = 0.152). Within this survivor group, patients with GOS 2 had the longest mean treatment duration (62.7 ± 43.6 days), followed by GOS 3 (38.7 ± 4.7 days), and GOS 5 (14 days, one patient with available data).

**Table 4:** Outcome analysis using chi-square test for different outcomes stratified by the GOS at discharge.

	Poor GOS 1–2 (*n* = 16)	Moderate GOS 3 (*n* = 5)	Fair GOS 4–5 (*n* = 4)	*p*
HH grade (median)	5	4	3	**0.012**
Fisher grade (median)	4	4	4	0.453
Treatment				0.143
- Clipping	8 (50%)	2 (40%)	4 (100%)	
- Endovascular	8 (50%)	3 (60%)	0	
Multiple aneurysms	5 (31.3%)	2 (40%)	0	0.369
New aneurysm formation	2 (12.5%)	0	0	0.543
Rebleeding	2 (12.5%)	0	0	0.543
Hydrocephalus with VP Shunt implantation	7 (43.8%)	2 (40%)	0	0.259
CVS	2 (12.5%)	1 (20%)	0	0.653
Pathogen detection and targeted antibiotic agent	5 (31.3%)	0	0	0.172
Mean antibiotic therapy duration (days)	40.8 (60.7*)	66 (90*)	14	N/A

HH = Hunt & Hess; GOS = Glasgow Outcome Scale; VP = ventriculoperitoneal; CVS = cerebral vasospasm.

*Mean antibiotic therapy duration without patients that died during the hospital stay.

We did not observe significant differences in GOS and treatment modality (Clipping vs. Endovascular, *p* = 0.063, Spearman’s correlation). Table 4 provides an overview to the patients’ outcome.

### Case presentation

**Fig. 4:**
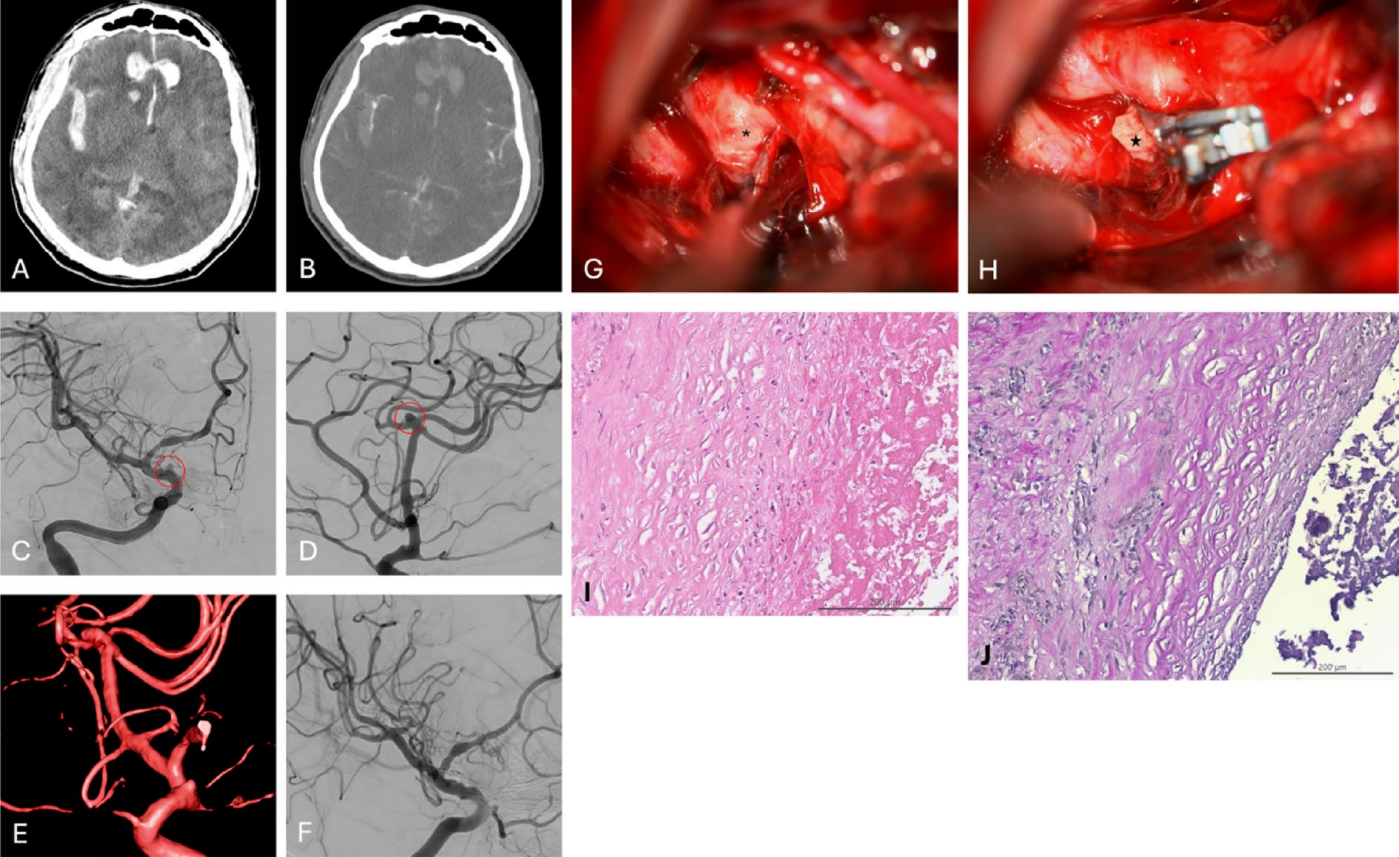
**(A**) Native CT and (**B**) CT angiography demonstrating an intracerebral hemorrhage and a right MCA aneurysm. (**C**) DSA identifies a small aneurysm of the terminal ICA and (**D**) an aneurysm at the trifurcation of the MCA. (**E**) 3D angiography with both aneurysms visible. (**F**) postoperative DSA shows successful clip elimination of both aneurysms with a dysplastic ICA with a suspected intraluminal thrombus. (**G**) Intraoperative photograph showing a dysplastic vessel wall of the ICA (*). (**H**) Clipping of the aneurysm with purulent thrombotic material revealed (★). This procedure requires special care due to the extreme thinning of the aneurysm wall caused by the inflammatory changes. (**I**) High-magnification H&E showing disintegration of the vessel wall and infiltration of lymphocytes and plasma cells. (**J**) EvG stained section of the aneurysm wall, displaying fragmentation of elastic fibers, as well as thinning of the media as a sign for the structural fragility of the aneurysm wall. This case highlights the complexity of multifocal mycotic aneurysms, demonstrating the importance of advanced imaging in diagnosing and planning the treatment of ruptured MIAs.

A 34-year-old male, originally from India, with no known pre-existing conditions or regular medication, experienced a sudden onset of a severe thunderclap headache followed by a tonic-clonic seizure. Upon arrival of the emergency medical team, the patient presented with GCS 3 requiring endotracheal intubation. During the transfer to our clinic, the patient developed anisocoria, which resolved after the EVD placement on admission. The initial CT scan revealed an aSAH with intraventricular hemorrhage classified as Fisher grade 4 (Fig. 4 and A + B). DSA subsequently identified two intracranial aneurysms: a 2 × 2 mm saccular elongation of the right distal carotid artery with an adjacent appositional thrombus appearing as a blister-like or partially thrombosed aneurysm, which was suspected as the source of the hemorrhage, and a 3.5 × 3 mm saccular aneurysm at the right MCA trifurcation, presumed to be unruptured (Fig. 4 and C + D). The patient underwent emergent hemicraniectomy and surgical clipping of both aneurysms. Intraoperative findings confirmed the MCA aneurysm as unruptured. Upon exposure of the right ICA, the entire parenting segment displayed an abnormal vessel wall appearance (Fig. 4 and G). The ICA aneurysm was occluded following placement of a temporary clip on the proximal ICA (Fig. 4 and H). After clipping, the aneurysm dome was opened, releasing purulent appearing material. Samples were collected for microbiological and histopathological analyses (Fig. 4 and I + J).

The postoperative DSA demonstrated complete occlusion of both aneurysms with persistent evidence of the intraluminal thrombus (Fig. 4 and E + F). A low-dose heparin infusion was initiated to prevent secondary enlargement of the thrombus. Given the intraoperatively observed vessel wall abnormalities, an interdisciplinary decision was made for early follow-up imaging, with consideration of additional stenting over the affected segment depending on the imaging results.

A repeat DSA performed four days later confirmed no residual filling of the clipped aneurysms, with regression of the thrombotic apposition and no progression of the aneurysmal outpouching of the intracranial ICA. However, the vessel wall continued to display extensive dysplastic changes. Additionally, a new five mm pseudoaneurysm of the left V3 segment, closely resembling a dissecting aneurysm, was observed (Supplementary Fig. 3C). Although this newly formed lesion was not explicitly classified as a mycotic aneurysm, its presence alongside rapidly progressive and widespread vascular abnormalities suggested that inflammatory vessel wall damage may have contributed to its formation. Due to the extent of the vascular involvement, the interdisciplinary team decided against stenting. Instead, the patient was maintained on heparin therapy, and systemic anti-infective treatment was initiated.

Microbiological workup revealed *C. acnes* in both blood cultures and intraoperative samples, while *Staphylococcus saccharolyticus* was identified in blood cultures only. Histological examination demonstrated a mixed thrombus, accompanied by abundant neutrophilic granulocytes, consistent with a diagnosis of MIA. The patient was treated with meropenem and vancomycin until Day 20, amounting to 35 days of antibiotic therapy post-SAH. On Day 31 after SAH, the patient underwent autologous bone flap cranioplasty on the right side and VP shunt placement on the left side. Postoperatively, a right-sided subdural hematoma was detected on a cranial CT scan. This was evacuated through a minor procedure without any neurological deterioration.

Despite extensive multimodal diagnostics, including urinalysis, chest x-ray, blood cultures, TTE and ENT examination, no underlying infectious source could be identified during the patient’s hospital course.

On day 46 after the SAH, the patient was discharged to a rehabilitation facility. At discharge, the patient was awake, alert and able to form short sentences. He showed spontaneous movement in all extremities, with more movement on the right side than on the left. The modified Rankin Scale (mRS) was 4 with a GOS of 3.

## Discussion

With this study, we aimed to provide a more comprehensive characterization of aSAH linked to the rupture of MIAs and to analyze microbiological and histopathological findings in connection with the following therapeutic strategies.

First, we aimed to characterize the clinical features accompanying aSAH due to the rupture of MIAs. Consistent with recent research, we found that patients frequently harbor multiple or may develop de-novo MIAs even within a short period during hospitalization^17,18^. Specifically, 20% of patients presented with more than one aneurysm on the initial DSA and further 16% developed new aneurysms during the same hospital stay in our cohort. This highlights the importance of close monitoring due to the increased risk of de-novo formation, rapid progression, and rupture of initially silent MIAs even under systemic treatment.

The risk of rebleeding observed in 12% in our series is likely tied to the dynamic of growth and infiltration and compromise of the arterial wall. In previous studies, it is reported to be even higher, ranging from 16% to 65% within the first month^17,20,21^. This emphasizes the need for early intervention and repeated imaging during hospitalization. Patients in our institutions typically undergo a DSA six months after aSAH if treated endovascularly or an MRI five years post-treatment if managed with surgical clipping. However, this approach should be adjusted for MIAs. In the present study, three patients (12%) developed new aneurysms during hospitalization with detection occurring between a few days and 2.5 months despite receiving appropriate antimicrobial therapy. Based on these findings, we recommend follow-up imaging using MR angiography within the first three months for patients who are discharged before this period.

The majority of aSAH (80%) in our cohort were classified as Fisher grade IV predominantly presenting as intracerebral hemorrhage (72%) at the site of the aneurysm. However, CVS occurred in only 8% of the cases (*n* = 2) likely due to the predominantly distal location of MIAs which are situated away from the brain cisterns. This site results in intraparenchymal hemorrhage rather than the typical aSAH patterns associated with cisternal involvement. Previous studies have shown that patients with aSAH due to ruptured aneurysms in the proximal anterior circulation are significantly more likely to develop CVS than those with aneurysms in more distal locations^22^. The most common location of MIAs in the literature is the MCA, which is consistent with our findings^17^.

Despite current treatment modalities, outcomes for patients with MIA remain poor. In our cohort, the mortality rate was 36% and the mean GOS at discharge was 2 indicating significant neurological impairment in survivors. These poor outcomes, including a GOS of less than 3 in a considerable number of patients (56%) reflect the challenges associated with the effective management of these cases.

As reported in recent literature^5^, the causative pathogens of MIAs are most often bacterial rather than fungal. Bacterial infections, particularly those associated with endocarditis, are a common cause, with organisms such as *Staphylococcus aureus* and *Streptococcus species* being most present, which is in line with our findings. Fungal infections are usually expected in immunocompromised patients. We detected fungal pathogens in three patients (12%) with irregular configured aneurysms located distally or at a vessel bifurcation and a Fisher grade IV bleeding on first CT with intracerebral hemorrhage. However, none of the three patients was immunosuppressed. These findings raise the question of why fungal pathogens appear in patients who are not usually considered to be immunocompromised.

In general, a global increase in the number of immunosuppressed patients is driven by medical advances and demographic changes^23,24^. More organ and stem cell transplants require immunosuppressive therapy^25^, while chemotherapy, radiotherapy and autoimmune treatments (e.g. TNF blockers, JAK inhibitors) also contribute. Immunosenescence characterized by a diminished immune response and an increased inflammatory activity is a key factor of aging-related immunosuppression^26^. In addition, HIV patients often have residual immune dysfunction despite Antiretroviral Therapy **(**ART)^27^. Chronic diseases such as diabetes, kidney disease, malnutrition and obesity also impair immunity and increase susceptibility to infection^28^. We propose that the increasing number of immunosuppressed patients will lead to a higher prevalence of endocarditis and mycotic (cerebral) aneurysms. Consequently, clinical awareness should be increased and standardized therapeutic treatment algorithms should be established.

In our cohort, infective endocarditis was the most common underlying infection, affecting 40% of patients. This finding is consistent with previous studies that identified endocarditis as a major contributor to MIA development: A retrospective study by Rice et al.. analyzed 618 patients with infective endocarditis who underwent cerebral angiography^29^. Forty patients in their cohort had 43 MIAs. They found that a longer duration of antibiotic treatment prior to the discovery of the MIA was significantly associated with a higher likelihood of aneurysmal regression or resolution (*p* = 0.046). However, their findings also emphasized that antibiotic therapy alone led to resolution in only 28% of cases, underscoring its limited efficacy as a standalone treatment. Furthermore, they highlight the dynamic nature of MIAs as many either remained unchanged, enlarged, or even ruptured despite treatment. Consequently, the study advocates for early detection and close monitoring to promptly identify high-risk aneurysms that may require intervention beyond conservative management. This also emphasizes earlier follow-up MRA imaging.

We observed a significant positive correlation between antibiotic treatment duration and GOS at discharge in the overall group with complete data (*n* = 18, *p* = 0.033). However, this association did not persist in the subgroup of survivors (*n* = 16, *p* = 0.152), where patients with poorer outcomes had received longer courses of therapy. These findings suggest that longer antibiotic treatment may reflect higher clinical complexity or longer survival, rather than causally contributing to a favorable neurological outcome. Thus, while prolonged treatment may be necessary in individual cases, its duration alone should not be interpreted as predictive of outcome. Although the median survival time among survivors was 42 days, our study was not designed to determine the optimal cut-off point. Therefore, prolonged therapy may be appropriate in complex cases, but it is essential to individualize treatment based on the patient’s clinical course and the characteristics of the pathogen. Although these findings provide some useful information, they should be interpreted with caution due to the presence of confounding clinical factors. Extensive blood culture diagnostics are useful in this context, along with the cultivation of potential pathogens from biopsies, combined with Polymerase Chain Reaction (PCR) for pathogen identification. As a novel diagnostic approach, next-generation sequencing (NGS) of cell-free DNA in the patient’s whole blood is a potential option^30,31^. To date, no studies have been conducted on this approach; however, it has proven beneficial in endocarditis.

Altogether, our findings highlight the need for further research into enhanced diagnostic and therapeutic strategies to improve outcomes for patients with MIAs. The rapid progression of aneurysms, the high mortality and the poor neurological outcomes suggest that current treatments may not be sufficient, particularly for patients who develop multiple or rapidly growing aneurysms.

### Limitations

This study has some limitations: First, the cohort size of only 25 patients is relatively small, which may limit the generalizability of our findings and does not provide sufficient statistical power to identify potential target factors for optimizing treatment and improving overall outcomes. The small sample size limited statistical power and prevented meaningful multivariable analyses. Therefore, our results remain exploratory and should be interpreted with caution. Given the low incidence of MIAs, a multicenter, multidisciplinary registry or collaborative study should be considered to gather larger datasets, facilitate robust statistical analysis, and develop evidence-based strategies for improved diagnosis, management and patient outcomes. Additionally, only six histopathological specimens were available for analysis out of the 25 patients included in the study, potentially restricting our ability to fully assess histological changes associated with MIAs.

Another key limitation of our study is the absence of prior vascular imaging in most cases before the onset of aSAH. As a result, although we defined MIAs as newly developed aneurysms in the context of infection, we could not confirm the de novo formation in all patients. It is possible that some aneurysms were pre-existing and only became clinically apparent due to infection-related vascular inflammatory processes.

Furthermore, the therapeutic approaches varied across cases due to the absence of standardized guidelines for these rare entities, resulting in individually tailored treatment concepts on a case-by-case basis. This variability in management introduces potential differences in outcomes, making it challenging to compare treatment efficacy systematically. Furthermore, in some cases no clear source of infection could be identified which may impact our ability to fully characterize the underlying infectious etiology and the appropriate therapy regimen in these patients. In addition, although the centers were intentionally selected for their similar infectious disease management and surgical approaches subtle differences in clinical practices and institutional protocols may still exist and may have influenced the results.

## Conclusion

Despite its rarity, aSAH due to the rupture of MIAs is associated with high mortality and poor functional outcomes. In our cohort, over one-third of patients presented with multiple or newly forming aneurysms, highlighting the dynamic and progressive nature of the condition. This underscores the need for early diagnosis, prompt aneurysm occlusion, broad-spectrum antimicrobial therapy, and follow-up imaging within the first weeks after diagnosis, as the patient’s initial clinical condition appears to be a critical determinant of prognosis.

While a positive correlation between antibiotic treatment duration and outcome was observed in the total cohort, subgroup analysis among survivors did not confirm this effect, suggesting that the association may be confounded by disease severity and survival duration.

Given the limited data, establishing a multicenter, multidisciplinary registry or collaborative study is essential to compile larger datasets, enable robust statistical analyses, and develop evidence-based strategies to improve patient outcomes.

## Supplementary Information

Below is the link to the electronic supplementary material.

Supplementary Material 1

## Data Availability

All source data are securely stored at the Department of Neurosurgery, Technical University of Munich. Researchers interested in data exchange are encouraged to reach out to the corresponding author directly.

## Declarations

### Generative AI and AI-assisted technologies in the writing process

During the preparation of this paper, the authors utilized ChatGPT4.0 (OpenAI, San Francisco, California, USA) to assist with spelling, grammar, and style corrections. Following the use of these tools, the authors thoroughly reviewed and made additional corrections to ensure accuracy and appropriateness. The authors take full responsibility for the final content and conclusions presented in this publication.
