# Supplementary material for: Mycotic aneurysms as a rare cause of subarachnoid hemorrhage
Source: Sci Rep. 2025 Sep 23;15:32683. doi: 10.1038/s41598-025-20673-8 (PMC12457590; doi:10.1038/s41598-025-20673-8)
Supplement: Supplementary file 1 — Supplementary Material 1 [file 41598_2025_20673_MOESM1_ESM.docx]

**Supplementary Material**

**
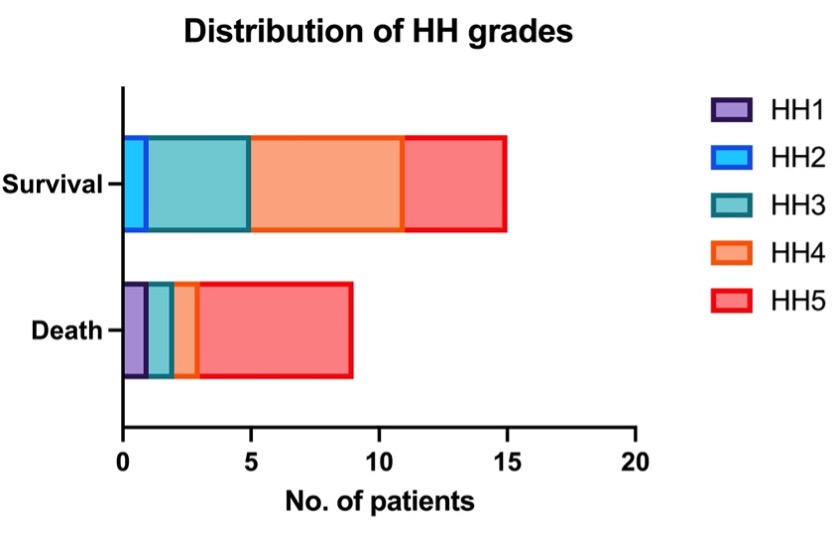
**

**Supplementary Figure 1** Distribution of HH grades at admission among surviving and deceased patients.


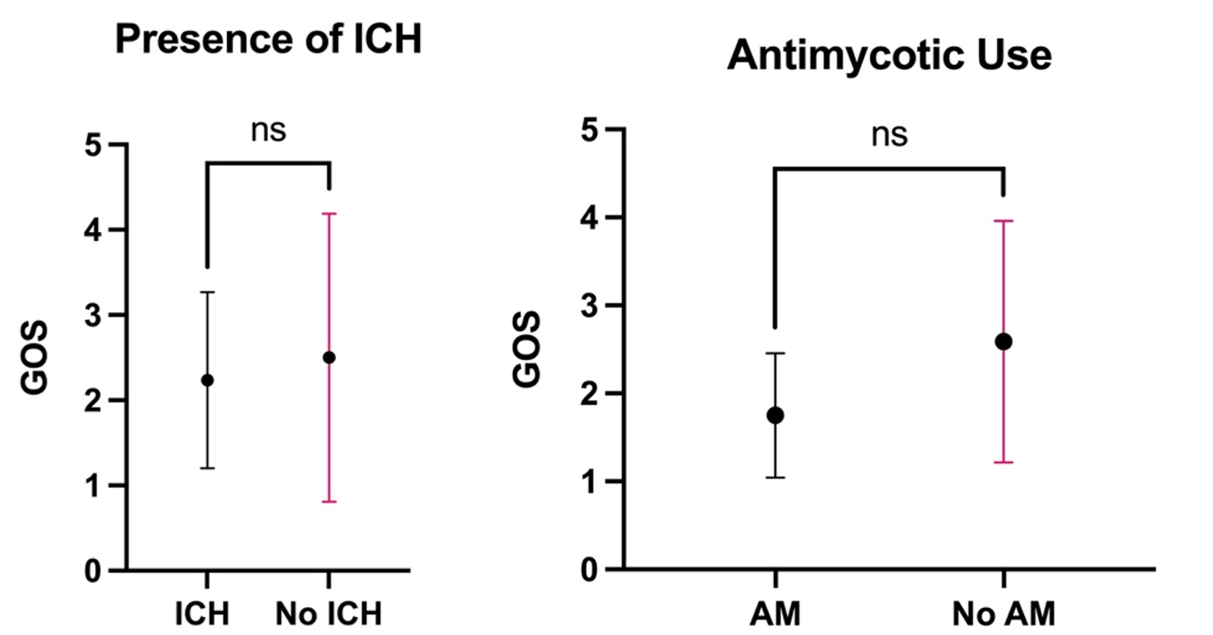


**Supplementary Figure 2** Neither the presence of an intracerebral hemorrhage nor the use of antimycotic agents correlated with GOS values at discharge.

**
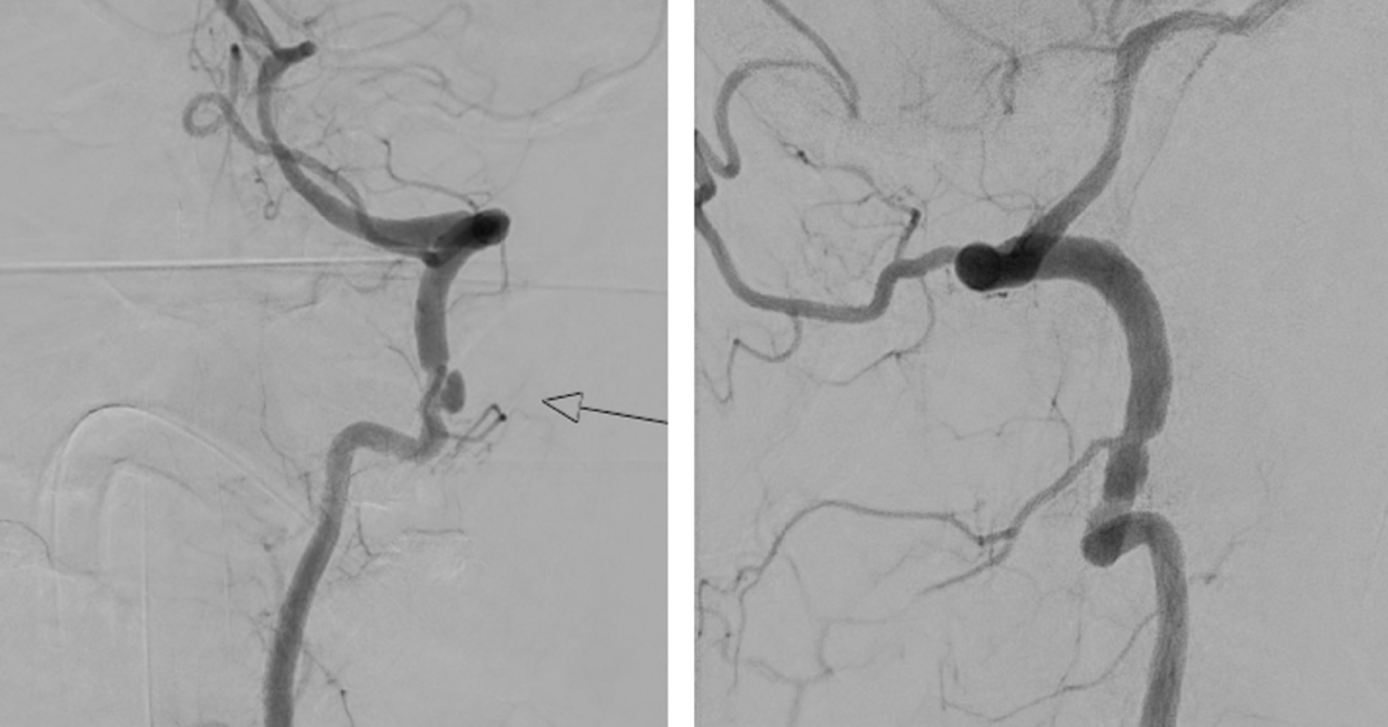
**

**Supplementary Figure 3** Postoperative DSA of the 34-year-old patient on postoperative day 4 shows a newly developed dissecting pseudoaneurysm (~5 mm) in the left V3 segment, with irregularities in the parent vessel. Although this lesion is not classified as an infectious aneurysm, it occurred in the context of widespread vascular wall changes and alongside a confirmed MIA of the right ICA. Its presence may reflect secondary vessel wall damage due to systemic or localized inflammatory processes.

**Supplementary Table 1.** Mode of pathogen detection in patients with *Cutibacterium acnes* (*C. acnes*).

*Patient from case report.

|  | **Suspected focus** | **Positive blood culture** | **Positive intraoperative sample** |
| --- | --- | --- | --- |
| **46 y/o male** | Prior intracranial surgery | *Candida parapsilosis* | *C. acnes* (epidural) |
| **43 y/o male** | Prior intracranial surgery | *Candida parapsilosis* | *C. acnes*, *Klebsiella pneumoniae* (CSF) |
| **34 y/o male*** | none | *C. acnes*, *S. saccharolyticus* | *C. acnes* (aneurysm tissue) |
| **74 y/o male** | none | - | *C. acnes* (abscess) |

**Supplementary Table 2.** Pathogen Spectrum and Immunosuppression Status in Patients with MIA and concomitant Endocarditis

| **Patient** | **Identified Pathogens** | **Immunosuppression Status** |
| --- | --- | --- |
| 1 | *Streptococcus anginosus*, *Candida albicans*, *Staphylococcus haemolyticus*, *Staphylococcus epidermidis* | No immunosuppression |
| 2 | *Candida albicans* | Intravenous drug use |
| 3 | *Staphylococcus haemolyticus* | pANCA-positive rheumatologic condition with immunosuppressive therapy |
| 4 | No pathogens detected | No immunosuppression |
| 5 | *Streptococcus pyogenes*, *Staphylococcus aureus* | Untreated HIV infection |
| 6 | *Staphylococcus epidermidis*, *Gemella haemolysans* | No immunosuppression |
| 7 | *Streptococcus mutans* | No immunosuppression |
| 8 | Methicillin-sensitive *Staphylococcus aureus* (MSSA) | HIV-positive, under treatment |
| 9 | *Streptococcus anginosus* | No immunosuppression |
| 10 | *Abiotrophica defectiva* | No immunosuppression |
| 11 | No pathogens detected | Untreated HIV infection |
